# Associations between adipose tissue volume and small molecules in plasma and urine among asymptomatic subjects from the general population

**DOI:** 10.1038/s41598-020-58430-8

**Published:** 2020-01-30

**Authors:** Lerina Otto, Kathrin Budde, Gabi Kastenmüller, Anne Kaul, Uwe Völker, Henry Völzke, Jerzy Adamski, Jens P. Kühn, Jan Krumsiek, Anna Artati, Matthias Nauck, Nele Friedrich, Maik Pietzner

**Affiliations:** 1 Institute of Clinical Chemistry and Laboratory Medicine, University Medicine Greifswald, Greifswald, Germany; 2 DZHK (German Center for Cardiovascular Research), partner site Greifswald, Greifswald, Germany; 3 Institute of Bioinformatics and Systems Biology, Helmholtz Zentrum München, Neuherberg, Germany; 4 Interfaculty Institute for Genetics and Functional Genomics, University Medicine and Ernst-Moritz Arndt-University Greifswald, Greifswald, Germany; 5 Institute for Community Medicine, University Medicine Greifswald, Greifswald, 17475 Germany; 6 DZD (German Center for Diabetes Research), site Greifswald, Greifswald, 17475 Germany; 7 Institute of Experimental Genetics, Genome Analysis Center, Helmholtz Zentrum München, Neuherberg, Germany; 8 Lehrstuhl für Experimentelle Genetik, Technische Universität München, Freising-Weihenstephan, Germany; 9 DZD (German Center for Diabetes Research), München-Neuherberg, Germany; 10 Institute of Diagnostic Radiology and Neuroradiology, University Medicine Greifswald, Greifswald, Germany; 11 Institute of Diagnostic Radiology, University Medicine, Carl Gustav Carus University Dresden, Dresden, Germany; 12 ICB (Institute of Computational Biology), Helmholtz Zentrum München, Neuherberg, 85764 Germany; 13 Institute for Computational Biomedicine, Englander Institute for Precision Medicine, Department of Physiology and Biophysics, Weill Cornell Medicine, New York, NY USA

**Keywords:** Metabolic syndrome, Metabolic syndrome

## Abstract

Obesity is one of the major risk factor for cardiovascular and metabolic diseases. A disproportional accumulation of fat at visceral (VAT) compared to subcutaneous sites (SAT) has been suspected as a key detrimental event. We used non-targeted metabolomics profiling to reveal metabolic pathways associated with higher VAT or SAT amount among subjects free of metabolic diseases to identify possible contributing metabolic pathways. The study population comprised 491 subjects [mean (standard deviation): age 44.6 yrs (13.0), body mass index 25.4 kg/m² (3.6), 60.1% females] without diabetes, hypertension, dyslipidemia, the metabolic syndrome or impaired renal function. We associated MRI-derived fat amounts with mass spectrometry-derived metabolites in plasma and urine using linear regression models adjusting for major confounders. We tested for sex-specific effects using interactions terms and performed sensitivity analyses for the influence of insulin resistance on the results. VAT and SAT were significantly associated with 155 (101 urine) and 49 (29 urine) metabolites, respectively, of which 45 (27 urine) were common to both. Major metabolic pathways were branched-chain amino acid metabolism (partially independent of insulin resistance), surrogate markers of oxidative stress and gut microbial diversity, and cortisol metabolism. We observed a novel positive association between VAT and plasma levels of the potential pharmacological agent piperine. Sex-specific effects were only a few, e.g. the female-specific association between VAT and O-methylascorbate. In brief, higher VAT was associated with an unfavorable metabolite profile in a sample of healthy, mostly non-obese individuals from the general population and only few sex-specific associations became apparent.

## Introduction

Obesity is one of the major risk factors for the development of chronic diseases like type 2 diabetes mellitus (T2DM), dyslipidemia or hypertension^1^ and therefore an accelerating cause of mortality^2^. Apart from overall obesity as measured by the body mass index (BMI) the specific site of excessive fat accumulation seems to be more important for disease risk. Accumulation of visceral adipose tissue (VAT) has been associated with metabolic and cardiovascular diseases (CVD)^3^ and is suspected to account for sex-specific differences in disease risks^4,5^. A more recent genetic approach has established a causal link between VAT and metabolic diseases with an astonishingly sex-difference, the odds for having T2DM being about 3-fold higher in women compared to men for the same absolute increase in VAT^6^. Nevertheless, in both sexes accumulation of VAT was strongly related to a higher risk of CVD including myocardial infarction or hypertension as well as mortality^7–9^. The role of subcutaneous adipose tissue (SAT) remains to be determined.

Previous studies took advantage of targeted and non-targeted metabolomic profiling to investigate molecular pathways associated with higher amounts of VAT and/or SAT identifying multiple associated small molecules, such as branched-chain amino acids (BCAAs), tryptophan catabolites, glutamate or different lipid species^10–18^. However, study populations were either small, the metabolomics techniques used had only restrictive coverage, e.g. only few dozen measured metabolites, or only one type of bio specimens was used for investigation. Comprehensive non-targeted profiling of plasma and urine samples using mass spectrometry has been shown to gain complementary insights into molecular pathways associated with diverse clinical relevant phenotypes^19^. The exclusion of participants with either manifest metabolic diseases, like T2DM, or those being at high risk, i.e. presenting with components of the metabolic syndrome or hypertension, reduces unwanted confounding when investigating VAT/SAT-associated molecular signatures in the metabolome of blood or urine samples^20^.

We present here a cross-sectional study on the associations between the amount of VAT/SAT and small molecules measured in plasma and urine of about 500 participants from the general population free of T2DM, the metabolic syndrome, hypertension, dyslipidemia or renal impairment. In doing so, we aimed to identify pathways probably involved in the translation of higher VAT or SAT into manifest metabolic disease and to contrast the metabolic signatures associated with higher fat accumulation at different sites.

## Results

Applying the just mentioned exclusion criteria 491 participants [mean (standard deviation): age 44.6 yrs (13.0), body mass index 25.4 kg/m² (3.6), 60.1% female] were available for statistical analysis. Sex-specific characteristics of the study population are shown in Table 1 indicating the anticipated sex-specific distribution of adipose tissue at subcutaneous (higher in women) and visceral (higher in men) sites (Supplemental Fig. S1). Women showed a more beneficial lifestyle as indicated by less smoking and less alcohol consumption, whereas men had higher blood pressure, BMI as well as a higher estimated glomerular filtration rate (eGFR). Only small differences in blood lipid levels became apparent.

**Table 1:** General characteristics by sex.

Characteristics	Men (n = 192)	Women (n = 299)	p
Age (years)	43 (33; 52)	45 (38; 56)	0.08
Smoking (%)			<0.01
never smokers	32	47	
former smokers	41	28	
current smokers	27	25	
Physically active (%)	77	76	0.26
Alcohol consumption (g/day)	7.3 (2.8; 17.7)	2.6 (0.7; 6.5)	<0.01
Waist circumference (cm)	87 (82; 94)	77 (71; 83)	<0.01
BMI (kg/m²)	25.6 (23.8; 28.0)	24.5 (22.3; 27.7)	<0.01
Visceral adipose tissue (l)	3.35 (1.90; 5.06)	1.64 (0.96; 2.76)	<0.01
Subcutaneous adipose tissue (l)	5.17 (3.73; 6.60)	7.02 (5.59; 9.42)	<0.01
HbA1c (%)	5.1 (4.8; 5.4)	5.0 (4.8; 5.4)	0.13
Glucose (mmol/l)	5.3 (4.9; 5.5)	5.0 (4.8; 5.3)	<0.01
Insulin (μU/ml)	6.7 (4.6; 8.9)	7.2 (5.6; 10.1)	<0.01
Total cholesterol (mmol/l)	5.2 (4.5; 5.9)	5.3 (4.7; 6.1)	0.02
HDL cholesterol (mmol/l)	1.35 (1.17; 1.55)	1.67 (1.43; 1.92)	<0.01
LDL-cholesterol (mmol/l)	3.28 (2.66; 3.87)	3.20 (2.58; 3.74)	0.26
Triglycerides (mmol/l)	1.11 (0.78; 1.42)	0.98 (0.72; 1.33)	0.01
eGFR (ml/min/1.72 m²)	94 (85; 110)	89 (78; 102)	<0.01
Systolic BP (mmHG)	122 (115; 129)	110 (105; 121)	<0.01
Diastolic BP (mmHG)	75 (70; 80)	72 (67; 77)	<0.01

Continuous data are expressed as median (25th percentile; 75th percentile); nominal data are given as percentages. *χ2-test (nominal data) or Mann-Whitney test (interval data) were performed. HbA1c = glycated hemoglobin, HDL = high density lipoprotein, LDL = low density lipoprotein, eGFR = estimated Glomerular Filtration Rate, BP = blood pressure, BMI = body mass index, All parameters were measured from fasting blood samples.

### Sex-specific associations

**Figure 1:**
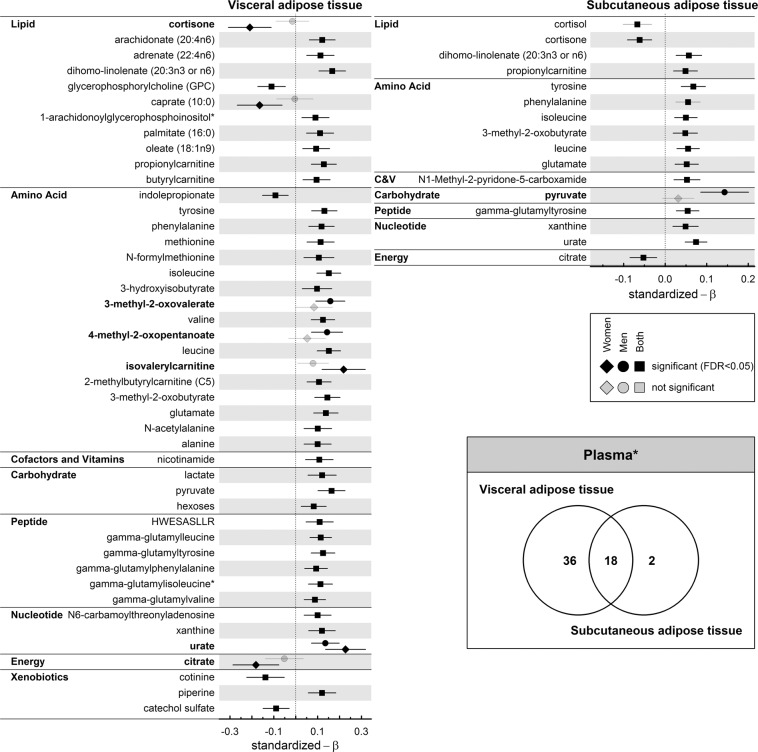
Standardized β-estimates from linear regression analyses with the amount of visceral (VAT; left panel) or subcutaneous (SAT; right panel) adipose tissue as exposure and plasma metabolites as outcome conducting either the whole population (square), only men (circle) or women (diamond). Displayed are only metabolites which were annotated and significant (controlling the false discovery rate (FDR) at 5%) in at least one of the subsets (indicated by darker colors). Metabolites printed in bold showed a nominal significant (p < 0.05) interaction term between VAT or SAT and sex. Regression models were adjusted for age, (sex), smoking behavior, alcohol consumption, LDL-cholesterol, systolic blood pressure, and estimated glomerular filtration rate. The Venn diagram displays the overlap in associated metabolites, including unknown(*) compounds.

**Figure 2:**
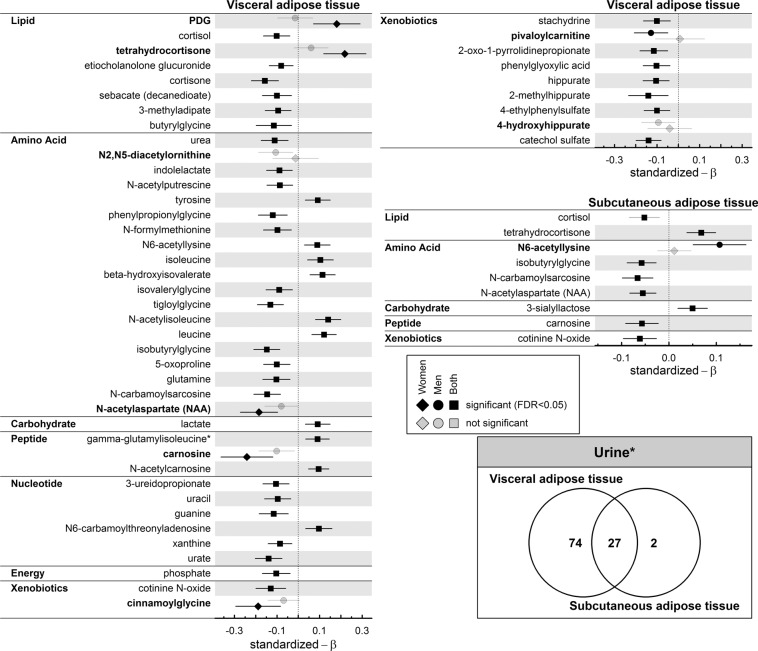
Standardized β-estimates from linear regression analysis with the amount of visceral (VAT; left panel) or subcutaneous (SAT; right panel) adipose tissue as exposure and urine metabolites as outcome conducting either the whole population (square), only men (circle) or women (diamond). Displayed are only metabolites which were annotated and significant (controlling the false discovery rate (FDR) at 5%) in at least one of the subsets (indicated by darker colors). Metabolites printed in bold showed a nominal significant (p < 0.05) interaction term between VAT or SAT and sex. Regression models were adjusted for age, (sex), smoking behavior, alcohol consumption, LDL-cholesterol, systolic blood pressure, and estimated glomerular filtration rate. The Venn diagram displays the overlap in associated metabolites, including unknown(*) compounds. PDG = 5beta-pregnan-3alpha,21-diol-11,20-dione 21-glucosiduronate.

In sex-interaction analyses 8 plasma and 19 urine metabolites showed at least a nominal significant interaction with VAT (Figs. 1, 2 and S1, 2). Associations unique to women in plasma included cortisone, caprate, citrate (all inversely) and isovalerylcarnitine (positively). The positive association with plasma urate had a stronger effect size in women compared to men, whereas the positive associations with the BCAA metabolites 3-methyl-2-oxovalerate and 4-methyl-2-oxopentoate were unique to men.

**Figure 3:**
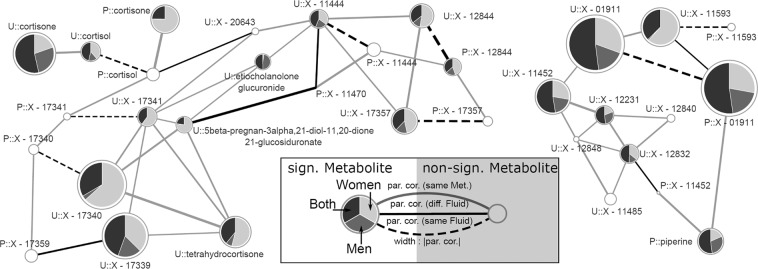
Subnetwork of the derived GGM with emphasize on cortisol as well as piperine and related compounds (e.g. X – 11593 putatively O-methylascorbate). On each node the results from linear regression analyses for visceral fat were mapped for the whole population (black), only women (light grey) or men (dark grey) as portion of the associations strength given as –log_10_(FDR-value). Significant results in at least one population, false discovery rate (FDR) below 5%, were highlighted by colors. Node sizes were chosen as maximum association strength. The prefix P denotes plasma metabolites whereas U indicates urine metabolites. Edges represent significant partial correlations (par. cor.) between metabolites. Type and color represent metabolite and fluid dependencies. Regression models were adjusted for age, (sex), smoking behavior, alcohol consumption, LDL-cholesterol, systolic blood pressure, and estimated glomerular filtration rate.

Unique positive associations regarding VAT and urine metabolites in women included several steroids, e.g. tetrahydrocrotisone and likely related unknown compounds as indicated from the estimated metabolic network (see Methods, Figs. 2, 3 and S2), as well as piperine along with related unknown compounds (Figs. 3 and S3). Inverse association between VAT and urine levels of, carnosine, N-acetylaspartate and cinnamoylglycine were unique to women as well. Among men only VAT and SAT were inversely associated with urine levels of pivaloylcarnitine and positively with urine levels of N6-acetyllysine,

### Consistent associations between the sexes

Linear regression analysis revealed 54 and 20 plasma metabolites to be associated with VAT and SAT, respectively, of which 18 were common to both (Fig. 1). This disproportional number of associated metabolites was even more obvious in urine as 101 metabolites significantly associated with VAT and 29 with SAT, 27 of which being common to both exposures (Fig. 2).

#### Plasma and urine metabolites associated with VAT

The majority of significant associations of VAT with the plasma metabolome were in positive direction comprising few lipid species (e.g. arachidonate, palmitate or butyrylcarntine), members of branched-chain amino acids (BCAA; e.g. valine or leucine) and related intermediates (e.g. 3-methyl-2-oxobutyrate), aromatic amino acids like tyrosine or phenylalanine as well as alanine and glutamate. Plasma levels of corresponding γ-glutamyl amino acids were positively associated as well. VAT was further positively associated with plasma levels of carbohydrates and related metabolites such as lactate, pyruvate and the sum of hexoses. Several unknown compounds were positively associated as well (Fig. S2) and visual inspection of the derived metabolic network revealed a cluster containing the annotated metabolite piperine (Fig. 3). Significant inverse associations were limited to glycerophosphocholine, cotinine and catechol sulfate (Fig. 1) as well as several unknown compounds (Fig. S2).

With respect to urine, inverse associations with VAT included steroids, like cortisol, and other fatty acids, e.g. sebacate, members of the urea cycle, e.g. urea itself, as well as purines and pyrimidines, like uracil, guanine or urate. Some of the associations seen in plasma, such as the positive associations with BCAAs, replicated in urine. However, urine levels of closely related catabolites of BCAAs, such as propionylglycine and more in general glycine-conjugated metabolites were inversely associated with VAT. Compared to these known metabolites an equal amount of unknown compounds showed significant associations with VAT, including those related to either cortisol or piperine in the derived metabolic network (Fig. 3).

#### Plasma and urine metabolites associated with SAT

Almost all association between SAT and metabolites were already described for VAT. Within each fluid, only two significant observations were unique to SAT (Figs. 1 and 2). Cortisol (inversely) and N1-methyl-2-pyridone-5-carboxamide (positively) levels in plasma as well as 3′-sialyllactose and the unknown compound X-12840 (both positively) levels in urine associated with SAT.

### Influence of insulin resistance

**Figure 4:**
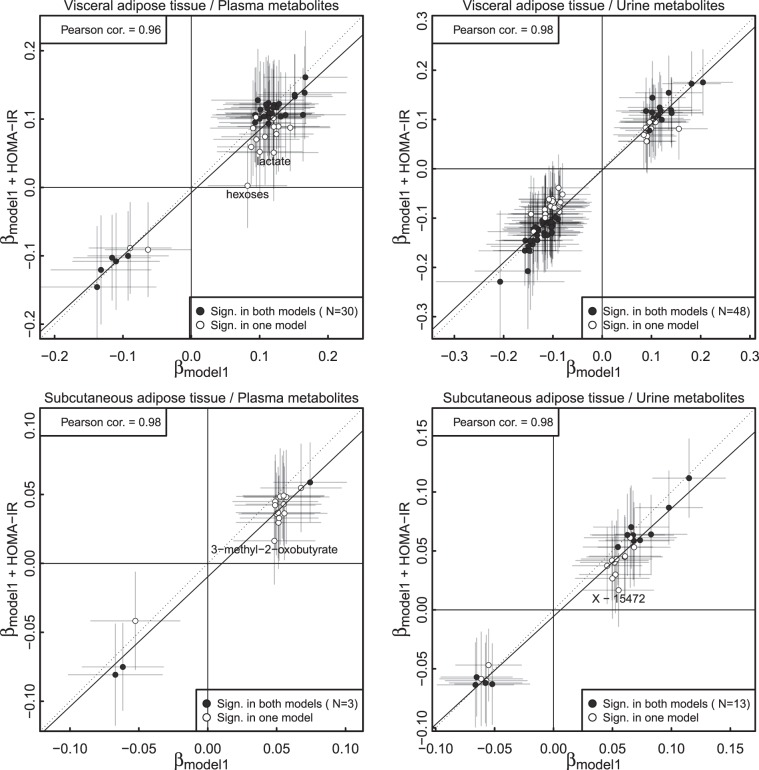
Comparison of the effect sizes (95%-CI indicated by lines) from linear regression models using visceral (VAT, upper panel) or subcutaneous (SAT, lower panel) adipose tissue and metabolite levels as outcome before (x-axis) and after (y-axis) further adjustment for the homeostatic model of insulin resistance (HOMA-IR). Model 1 was adjusted for age, sex, smoking behavior, alcohol consumption, low-density liporotein cholesterol, systolic blood pressure, and estimated glomerular filtration rate. Metabolites meeting statistical significance in both models (false discovery rate <5%) are indicated by darker colors and the number is given in brackets. Metabolites with strong attenuation of effect sizes (>50%) have been annotated. The solid line indicates the fit of an ordinary linear regression model between effect estimates from both models. The dotted line would indicate identity of effect estimates.

Inclusion of HOMA-IR in linear regression models did not change significant associations between VAT and plasma and urine levels of 30 and 48 metabolites, respectively, when conducting sex-pooled analysis (Fig. 4). Those included BCAAs (but not their primary degradation products, α-keto acids) and aromatic amino acids, gamma-glutamyl amino acids, glutamate, ω6-polyunsaturated fatty acids or palmitate in plasma as well as BCAA-catabolites, benzoate metabolites and cortisol metabolites in urine. Strong changes in effect sizes (>50%) were seen for the association between VAT and plasma levels of hexoses as well as lactate (Fig. 4). Three and 13 metabolites in plasma and urine, respectively, remained significantly associated with SAT when conducting sex-pooled analysis. Effect sizes for SAT on plasma 3-methyl-2-oxobutyrate and the urine unknown compound X-15472 were strongly attenuated following adjustment for HOMA-IR (Fig. 4).

The sex-specific effects observed for VAT and the plasma metabolites cortisone, X-12104, 3-methyl-2-oxovalerate, and urate remained unaffected after adjusting for HOMA-IR, similar as those for the urine metabolites tetrahydrocortisone, N-acetylaspartate, carnosine or several unknown compounds such as X-17340 or X-17357.

## Discussion

We used comprehensive non-targeted metabolomics profiling of two complementary body fluids to investigate molecular pathways associated with higher amounts of visceral and subcutaneous adipose tissue among a large sample of asymptomatic subjects from the general population. In the absence of metabolic disorders higher VAT was associated with molecular signatures previously implicated in the onset of T2DM and cardiovascular diseases such as impaired BCAA catabolism^21^, surrogates of oxidative stress^22^ and diminished gut microbial diversity^23^, and further with the potential pharmacological agent piperine and related catabolites. We observed only minor evidence for sex-specific associations, i.e. different effect sizes between the sexes for the same amount of increase in either VAT or SAT. With the exception of plasma cortisol, molecular signatures observed with respect to SAT were almost completely included in those seen with VAT.

### VAT is associated with BCAA metabolites independently of insulin resistance

Cross-sectional associations between VAT content and plasma BCAAs or related catabolites have been shown among obese subjects with a high burden from multiple diseases, including insulin resistance, and plasma concentrations of those metabolites were further shown to be lower among lean compared to (severely) obese subjects^18,24–27^. We observed a positive association between primary BCAAs as well as aromatic amino acids with VAT and SAT among subjects free of T2DM or the metabolic syndrome. Moreover, the association with VAT but not SAT persisted upon adjustment for HOMA-IR as a marker of peripheral insulin resistance. This observation might indicate that the association seen with SAT is only secondary to VAT as subjects with high amount of SAT also tend to have higher amounts of VAT. We observed some evidence that the association with downstream catabolites of BCAAs, such as 3-methyl-2-oxovalerate or 4-methyl-2-oxopentoate and VAT were stronger in men compared to women but was attenuated upon adjustment for HOMA-IR. Higher plasma concentrations of BCAAs have been consistently and repeatedly associated with higher risk of T2DM and other metabolic disorders independent of classical risk factors^21,26,28^. A common hypothesis implies impaired degradation of isoleucine, leucine, and valine in peripheral tissues, such as skeletal muscle, as responsible mechanism. The subsequent accumulation of α-keto acids, like 3-methyl-2-oxobutyrate, may trigger the pathophysiology of insulin resistance and subsequently T2DM with support from animal models^29^ and causal assessment using Mendelian randomization^30^. A recent study using elegant tracer studies in humans and mice revealed a so far unexpected role of brown adipose tissue in whole body catabolism of BCAAs^31^. Given the inverse correlation between VAT and brown adipose tissue mass^32^ one might speculate that this mechanism contributed to our observations as well. Increased BCAA availability, irrespective of the source (e.g. protein breakdown or nutrition^29^) might fuel other metabolic pathways including gluconeogenesis upsetting glucose homeostasis. In general, this molecular signature fits to the independent role of visceral obesity in the progression of insulin resistance and T2DM^33,34^ but requires further investigation about ‘the first hit’ leading to either higher VAT or BCAA accumulation in plasma.

### VAT associates with surrogates of (hepatic) oxidative stress

Besides the metabolism of BCAAs, the present study associated visceral obesity with an accumulation of closely related γ-glutamyl-dipeptides in plasma consistently across the sexes^35^. The formation of γ-glutamyl-dipeptides is part of the γ-glutamyl cycle, among others responsible for the restoration of the intracellular anti-oxidant glutathione. One previous metabolomics study^22^ suggested γ-glutamyl-dipeptides as sensitive markers for liver function, ranging from simple steatosis to hepatocellular carcinoma. The relation to non-alcoholic fatty liver disease was replicated by others^36^ and ectopic accumulation of fat within the liver is a condition frequently linked to visceral obesity^37^ and hence might relate our observations with impaired liver metabolism. Excess intake of food rich in calories and the subsequent availability of fuel likely induce increased mitochondrial substrate oxidation resulting in increased production of reactive oxygen species (ROS). ROS in turn stimulates glutathione production via NRF2 signaling and subsequent increased expression of γ-glutamylcysteine ligase, the key enzyme in glutathione production^38^. Subsequently, an accumulation of γ-glutamyl-dipeptides would indicate increased (hepatic) oxidative stress, as has been reported previously^22^. However, in spite of only slightly altered plasma fatty acid profiles the metabolically benign state of the participants might indicate a still adequate handling of the fuel excess.

### The black pepper ingredient and potent pharmacological agent piperine associates with VAT

Data-driven reconstruction of a metabolic network enabled us to reveal a cluster of metabolites positively associated with VAT and SAT which included the annotated plasma metabolite piperine (Fig. 3). The strongest associations with VAT, however, were seen for plasma and urine levels of the unknown compound X-01911. Such an enriched cluster might be indicative for an association with the whole pathway of piperine metabolism. Piperine can be derived from the degradation of black pepper^39^ and has been suggested as putative pharmacological agent for different applications^40,41^. With respect to adipose tissue distribution, a study in mice suggested a body fat lowering effect^42^ and piperine-treatment of 3T3-L1 cells reduced lipid storage as well as blocked their differentiation into adipocytes^39^. However, these observation contrasts our finding of a positive association between VAT and plasma piperine. One might speculate that the frequently described beneficial effects of piperine^43^ might have contributed to the metabolically healthy state of our study population with respect to major disease traits despite a high amount of VAT/SAT. A similar argument has been stated in traditional Chinese medicine^44^.

### VAT is associated with circulating surrogates of diminished gut microbial diversity

Microbial metabolism in the gut contributes to a significant amount to the metabolome of plasma and urine^45,46^. One of the most consistently reported surrogate metabolites is hippurate^23^. Urine hippurate levels, along with other putative metabolites of microbial origin such as indolepropionate (plasma) or 2-methylhippurate (urine), were inversely associated with VAT consistently in both sexes. These metabolites likely result from microbial digestion of dietary fibers, phenols or choline^23,46^. Several studies (for review see^23^) already reported associations between hippurate and obesity as well as T2DM. Obesity in general has been associated with a diminished alpha-diversity of the gut microbiome^47^ reflecting a loss of plurality in microbial species. By the use of triangulation among diet intake, visceral fat mass and gut microbial profiling in the Twins-UK study the authors suggested plasma levels of hippurate as a putative link between dietary habits and adipocyte function with neuroglobin expression as putative candidate regulator^14^. Higher relative abundancies of the phenol degrading *Eubacterium dolichum* was the single microbial species linking all levels, i.e. diet score, visceral fat mass and plasma hippurate levels. Apart from shifts in the gut microbial composition other explanations of our observations might be considered as well. Despite the adjustment for kidney function in regression models using estimated GFR as a proxy an effect of altered kidney function could not be ruled out entirely. Diminished tubular secretion in obese subjects, which is not necessarily directly related to the GFR^48,49^, of hippurate and cinnamoylglycine might have accounted for our findings independent of gut microbial metabolism^49^.

### The female-specific VAT association with O-methylascorbate might indicate a common genetic architecture

One of the few metabolic signatures with indication of a sex-specific effect was the positive association with VAT/SAT and urine levels of X-11593, meanwhile identified as O-methylascorbate^50^. O-methylascorbate is a product of ascorbate (vitamin C) by O-methylation^51^ and one might speculate about higher catechol-O-methyltransferase (COMT) activity and therefore more degradation products in women with higher VAT. Single nucleotide variants (SNV) mapping to *COMT*, the gene encoding COMT, have been associated with food choices including a favour for unhealthy food^52^ but early genetic studies using similar variants at *COMT* failed to show an association with BMI or weight^53^. The latter might be likely due to limited statistical power since more recent genome-wide association analysis using data from UK Biobank (N > 300k) showed significant associations (p < 5e-8, retrieved via www.phenoscanner.medscl.cam.ac.uk^54^ on 15/09/2019) with several refined anthropometric traits, in particular measures of fat-free mass, with the exact same missense SNV (rs4680) as has been used to annotate X-11593 as O-methylascorbate. However, neither the *COMT* locus nor the SNV appeared to be significant when using predicted VAT mass as an outcome in a genome-wide association study^6^. To conclude, our female-specific observation of VAT-associated levels of O-methylascorbate maybe explainable by a common genetic architecture of anthropometric traits and intermediary metabolism but further studies are needed to disentangle the relationship in more detail.

### SAT compared to VAT shows sex-consistent associations with cortisol metabolites

Plasma and urine cortisol levels were consistently associated with SAT in both sexes whereas degradation products such as cortisone (plasma), tetrahydrocortisone (urine) as well as likely related unknown compounds (Fig. 3) were associated with VAT only in women. Such a sex-specific segregation of associated metabolites across this pathway might be explained by a switch in cortisol clearance among women with high VAT. Increased clearance of cortisol despite unaltered plasma levels has already been described for (abdominal) obese subjects and hyperactivity of the hypothalamic-pituitary-adrenal (HPA) axis suggested as possible explanation^55–57^. A key enzyme in the conversion of cortisone to cortisol is 11β-hydroxysteroid dehydrogenase (11β-HSD) but its expression levels in adipose tissue are uncertain. Early work suggested high 11β-HSD expression in omental (i.e. visceral) but not subcutaneous adipose tissue^58^, bypassing the HPA-axis due to local cortisol production from inactive cortisone. The latter would result in tissue-dependent hypercortisolism. More recent work^59^ provided evidence for higher cortisol secretion from SAT rather than VAT in humans. The secretion site might be of particular importance, as cortisol released from VAT is suspected to contribute to hepatic insulin resistance whereas cortisol released from SAT might be counteracted by the HPA-axis^59^. It is important to note, however, that we observed inverse associations for plasma cortisol levels with SAT which is counterintuitive to the literature presented so far and might be attributable to the exclusion of subjects with obvious metabolic complications such as the metabolic syndrome or T2DM.

### Strengths and limitations

A clear strength of our study is the precise quantification of SAT and VAT using whole body magnetic resonance imaging in combination with a non-targeted MS-based metabolomics approach covering a huge diversity of small molecules from different origin. As we used a cross-sectional approach it is difficult to determine the causal nature of the associations presented and results should be interpreted with caution. Furthermore, dietary differences among subjects, possibly affecting the measured metabolome, could not be addressed adequately due to the missing of appropriate questionnaires.

## Conclusion

Among asymptomatic subjects higher amounts of VAT in both men and women were associated with several molecular signatures which have been previously implied in the onset of metabolic and cardiovascular disorders such as T2DM and those metabolites or pathways can now be tested as intermediate factors. Only few sex-specific associations appeared and larger studies are needed to test for more subtle differences between the sexes and to confirm our results.

## Methods

### Study population

The Study of Health in Pomerania (TREND) (SHIP-TREND) is a second cohort of a population-based research project in West Pomerania, a rural region in north-east Germany^60^. A stratified random sample of 8826 adults aged 20–79 years was drawn from population registries. Sample selection was facilitated by centralization of local population registries in the Federal State of Mecklenburg-West Pomerania. Stratification variables were age, sex and city/county of residence. Baseline examinations were conducted between 2008 and 2012. Out of all invitations 4420 chose to participate (50.1% response). All participants gave written informed consent before taking part in the study. The study was approved by the ethics committee of the University of Greifswald and conformed to the principles of the declaration of Helsinki.

For a subsample of up to 1000 subjects without self-reported diabetes plasma as well as urine metabolomics data based on mass spectrometry (see below) were available. The analyses focused on asymptomatic subjects. Therefore, 455 subjects with hypertension, metabolic syndrome or an estimated glomerular filtration rate (eGFR) <50 ml/min/m^2^ were excluded. After further exclusion of subjects with missing values in the exposure variables the final study population comprised 491 subjects (192 men; 299 women).

### Measurements

Participants’ characteristics and medical histories were recorded using computer-aided personal interviews. Smoking status was categorized as current, former or never smokers. Mean daily alcohol consumption was calculated using beverage-specific pure ethanol volume proportions. Subjects who participated in physical training for at least two hours a week were classified as physically active. Waist circumference (WC) was measured to the nearest 0.1 cm using an inelastic tape midway between the lower rib margin and the iliac crest in the horizontal plane. Height was measured to the nearest 1 cm using a digital ultrasound instrument, and weight was measured using standard digital scales to the nearest 0.1 kg with the subject in light clothing and without shoes. Body mass index was calculated as kg/m². Hypertension was present by either an increased blood pressure (BP) (systolic BP of ≥140 mm Hg or a diastolic BP of ≥90) or the use of antihypertensive medication (self-report). Dyslipidemia was defined as follows: concentrations of total cholesterol ≥5.2 mmol/l (≥200 mg/dl), or low-density lipoprotein cholesterol (LDL) ≥3.4 mmol/l (≥130 mg/dl), or high-density lipoprotein cholesterol (HDL) <1.04 mmol/l (<40 mg/dl), or use of anti-lipidemic medication. Metabolic syndrome (MetS) was defined by three or more of the following five components^61,62^ using fasting blood samples: (1) abdominal obesity: men WC ≥94 cm, women WC ≥ 80 cm; (2) elevated triglycerides: ≥2.3 mmol/l (fasting time <8 h) or ≥1.7 mmol/l (fasting time ≥8 h) or lipid-modifying medication (ATC code C10AB or C10AD); (3) reduced high-density lipoprotein (HDL) cholesterol: men <1.03 mmol/l, women <1.29 mmol/l; (4) elevated blood pressure: ≥130/85 mmHg or self-reported antihypertensive medication or (5) elevated glucose: ≥6.1 mmol/l or diabetic medication (ATC code A10).

Fasting blood samples (≥8 hours) were drawn between 7:00 am and 11:00 am from the cubital vein of subjects in the supine position and analyzed immediately or stored by −80 °C. Total cholesterol levels were measured by photometry (Dimension VISTA, Siemens Healthcare Diagnostics, Eschborn, Germany). HDL/LDL cholesterol levels were selectively precipitated and then determined by homogenous assays (Dimension VISTA, Siemens Healthcare Diagnostics, Eschborn, Germany). Plasma insulin levels were measured (Centaur XP by Siemens Healthcare Diagnostics) and the homeostatic model assessment of insulin resistance (HOMA-IR) index was calculated as insulin (μU/mL) × glucose (mmol/L)/22.5. Serum creatinine levels were measured using an enzymatic assay (Dimension VISTA, Siemens Healthcare Diagnostics, Eschborn, Germany). The eGFR was calculated as: eGFR = 186.3 × (serum creatinine)^−1.154^ × (age)^−0.203^ × (0.742 if female).

### Magnetic resonance imaging

Whole body magnetic resonance imaging (MRI) was performed on commercial 1.5-Tesla system (Magnetom Avanto, Siemens Healthcare AG, Erlangen, Germany, software version Syngo MR B15), using a body phased array coil. The quantification of subcutaneous and visceral fat was done using the automatic tissue and labeling analysis software ATLAS and an in-house developed software from the University of Ulm^63^. Afterwards a manual correction by certified students was applied.

### Metabolomics measurements

Non-targeted metabolomics analysis for metabolic profiling was conducted at the Genome Analysis Center, Helmholtz Zentrum München. A detailed description of metabolite measurements, annotations and data processing is given elsewhere^64,65^ and in the Supplemental Information. After preprocessing 475 plasma and 558 urine metabolites remained for the statistical analyses. Note that 177 plasma metabolites and 302 urine metabolites could not be unambiguously assigned to a chemical identity and are referred to hereafter with the notation “X” followed by a unique number. To estimate Gaussian graphical models (GGMs) based on metabolite data, missing values were imputed for metabolites with less than 20% missing values using sampling from truncated log-normal distributions and multiple imputations by chained equations afterwards.

### Statistical analysis

For bivariate analyses, the Kruskal-Wallis test (continuous data) or χ^2^ test (nominal data) were used to compare women and men. Linear regression models were performed to test the association between VAT as well as SAT (independent) and plasma as well as urine metabolites (dependent). Metabolite levels were rescaled to mean of zero and standard deviation of one. Since both fat compartments strongly differed between the sexes (Table 1) all analyses were performed including an interaction term with sex. If this revealed an at least nominal significant interaction (p < 0.05) models were estimated for each sex separately. If not otherwise noted, all models were adjusted for age, smoking, physical activity, alcohol consumption, systolic blood pressure, LDL cholesterol and eGFR. A second model further adjusting for HOMA-IR was used to test for the influence of insulin resistance on the presented results. To account for multiple testing, we corrected the p-values from regression analyses by controlling the false discovery rate (FDR) at 5% using the Benjamini-Hochberg procedure^66^. GGMs for the metabolome data were calculate because of their ability to mirror physiological dependencies^67^. An extensive description of the procedure could be found in the Supplemental Information. Briefly, GGMs rely on full-order partial correlations therefore a correlation between two metabolites only exists if it is independent from all remaining metabolites in the data set. Significant partial correlations after Bonferroni correction were visualized as network using Cytoscape 3.2.1. Statistical analyses were performed using R 3.5.2 (R Foundation for statistical computing, Vienna, Austria).

## Supplementary information

Supplementary Methods and Figures.

Dataset 1.

## Data Availability

SHIP data are publicly available for scientific and quality control purposes. Data usage can be applied for via www.community-medicine.de.

## References

[1] Daniels SR (2005). Overweight in children and adolescents: pathophysiology, consequences, prevention, and treatment. Circulation.

[2] Solomon, C. G. & Manson, J. E. Obesity and mortality: a review of the epidemiologic data. *Am J Clin Nutr* **66**, 1044S-1050S (1997). 10.1093/ajcn/66.4.1044S 9322585

[3] Grundy SM (2004). Definition of metabolic syndrome: report of the National Heart, Lung, and Blood Institute/American Heart Association conference on scientific issues related to definition. Arterioscler. Thromb. Vasc. Biol..

[4] Zhang Y (2014). Fat cell size and adipokine expression in relation to gender, depot, and metabolic risk factors in morbidly obese adolescents. Obes..

[5] White UA, Tchoukalova YD (2014). Sex dimorphism and depot differences in adipose tissue function. Biochim. Biophys. Acta.

[6] Karlsson T (2019). Contribution of genetics to visceral adiposity and its relation to cardiovascular and metabolic disease. Nat. Med..

[7] Koster A (2015). Fat distribution and mortality: the AGES-Reykjavik Study. Obes..

[8] Kuk JL (2006). Visceral fat is an independent predictor of all-cause mortality in men. Obes. 14, 336-341.

[9] Palmer BF, Clegg DJ (2015). The sexual dimorphism of obesity. Mol. Cell Endocrinol..

[10] Baek SH (2017). Metabolites distinguishing visceral fat obesity and atherogenic traits in individuals with overweight. Obes..

[11] Boulet MM (2015). Alterations of plasma metabolite profiles related to adipose tissue distribution and cardiometabolic risk. Am. J. physiology. Endocrinol. Metab..

[12] Maltais-Payette I, Boulet MM, Prehn C, Adamski J, Tchernof A (2018). Circulating glutamate concentration as a biomarker of visceral obesity and associated metabolic alterations. Nutr. Metab..

[13] Menni C (2016). Metabolomic profiling to dissect the role of visceral fat in cardiometabolic health. Obes..

[14] Pallister T (2017). Untangling the relationship between diet and visceral fat mass through blood metabolomics and gut microbiome profiling. Int. J. Obes..

[15] Rietman A (2016). Associations between plasma branched-chain amino acids, beta-aminoisobutyric acid and body composition. J. nutritional Sci..

[16] Scherer M (2015). Blood plasma lipidomic signature of epicardial fat in healthy obese women. Obes..

[17] Takashina C (2016). Associations among the plasma amino acid profile, obesity, and glucose metabolism in Japanese adults with normal glucose tolerance. Nutr. Metab..

[18] Yamakado M (2012). Plasma amino acid profile is associated with visceral fat accumulation in obese Japanese subjects. Clin. Obes..

[19] Pietzner M (2017). Comprehensive metabolic profiling of chronic low-grade inflammation among generally healthy individuals. BMC Med..

[20] Franks PW, Atabaki-Pasdar N (2017). Causal inference in obesity research. J. Intern. Med..

[21] Wang TJ (2011). Metabolite profiles and the risk of developing diabetes. Nat. Med..

[22] Soga T (2011). Serum metabolomics reveals gamma-glutamyl dipeptides as biomarkers for discrimination among different forms of liver disease. J. Hepatol..

[23] Lees HJ, Swann JR, Wilson ID, Nicholson JK, Holmes E (2013). Hippurate: the natural history of a mammalian-microbial cometabolite. J. Proteome Res..

[24] Green CR (2016). Branched-chain amino acid catabolism fuels adipocyte differentiation and lipogenesis. Nat. Chem. Biol..

[25] Menni C (2013). Biomarkers for type 2 diabetes and impaired fasting glucose using a nontargeted metabolomics approach. Diabetes.

[26] Newgard CB (2009). A branched-chain amino acid-related metabolic signature that differentiates obese and lean humans and contributes to insulin resistance. Cell Metab..

[27] Moore SC (2014). Human metabolic correlates of body mass index. Metabolomics.

[28] Tai ES (2010). Insulin resistance is associated with a metabolic profile of altered protein metabolism in Chinese and Asian-Indian men. Diabetologia.

[29] Lynch CJ, Adams SH (2014). Branched-chain amino acids in metabolic signalling and insulin resistance. Nat. reviews. Endocrinol..

[30] Lotta LA (2016). Genetic Predisposition to an Impaired Metabolism of the Branched-Chain Amino Acids and Risk of Type 2 Diabetes: A Mendelian Randomisation Analysis. PLoS Med..

[31] Yoneshiro T (2019). BCAA catabolism in brown fat controls energy homeostasis through SLC25A44. Nat..

[32] Brendle C (2018). Correlation of Brown Adipose Tissue with Other Body Fat Compartments and Patient Characteristics: A Retrospective Analysis in a Large Patient Cohort Using PET/CT. Academic radiology.

[33] Mathieu P, Boulanger MC, Despres JP (2014). Ectopic visceral fat: a clinical and molecular perspective on the cardiometabolic risk. Rev. Endocr. Metab. Disord..

[34] Van Gaal LF, Mertens IL, De Block CE (2006). Mechanisms linking obesity with cardiovascular disease. Nat..

[35] Bridges RJ, Meister A (1985). gamma-Glutamyl amino acids. Transport and conversion to 5-oxoproline in the kidney. J. Biol. Chem..

[36] Dumas ME, Kinross J, Nicholson JK (2014). Metabolic phenotyping and systems biology approaches to understanding metabolic syndrome and fatty liver disease. Gastroenterology.

[37] Byrne CD (2013). Ectopic fat, insulin resistance and non-alcoholic fatty liver disease. Proc. Nutr. Soc..

[38] Wild AC, Moinova HR, Mulcahy RT (1999). Regulation of gamma-glutamylcysteine synthetase subunit gene expression by the transcription factor Nrf2. J. Biol. Chem..

[39] Park UH (2012). Piperine, a component of black pepper, inhibits adipogenesis by antagonizing PPARgamma activity in 3T3-L1 cells. J. Agric. Food Chem..

[40] Tang Xin, Drotar Jesse, Li Keji, Clairmont Cullen D., Brumm Anna Sophie, Sullins Austin J., Wu Hao, Liu Xiaoxiao Shawn, Wang Jinhua, Gray Nathanael S., Sur Mriganka, Jaenisch Rudolf (2019). Pharmacological enhancement of KCC2 gene expression exerts therapeutic effects on human Rett syndrome neurons and Mecp2 mutant mice. Science Translational Medicine.

[41] Ma ZG (2017). Piperine Attenuates Pathological Cardiac Fibrosis Via PPAR-gamma/AKT Pathways. EBioMedicine.

[42] Okumura Y, Narukawa M, Watanabe T (2010). Adiposity suppression effect in mice due to black pepper and its main pungent component, piperine. Biosci. Biotechnol. Biochem..

[43] Derosa G, Maffioli P, Sahebkar A (2016). Piperine and Its Role in Chronic Diseases. Adv. Exp. Med. Biol..

[44] Zhang WL, Zhu L, Jiang JG (2014). Active ingredients from natural botanicals in the treatment of obesity. Obes. reviews: an. Off. J. Int. Assoc. Study Obes..

[45] Wilmanski T (2019). Blood metabolome predicts gut microbiome alpha-diversity in humans. Nat. Biotechnol..

[46] Nicholson JK (2012). Host-gut microbiota metabolic interactions. Sci..

[47] Wen Li, Duffy Andrew (2017). Factors Influencing the Gut Microbiota, Inflammation, and Type 2 Diabetes. The Journal of Nutrition.

[48] Suchy-Dicey AM (2016). Tubular Secretion in CKD. J. Am. Soc. Nephrology: JASN.

[49] Rivara MB (2017). Diurnal and Long-term Variation in Plasma Concentrations and Renal Clearances of Circulating Markers of Kidney Proximal Tubular Secretion. Clin. Chem..

[50] Krumsiek J (2012). Mining the unknown: a systems approach to metabolite identification combining genetic and metabolic information. PLoS Genet..

[51] Blaschke E, Hertting G (1971). Enzymic methylation of L-ascorbic acid by catechol O-methyltransferase. Biochem. Pharmacol..

[52] Wallace DL (2015). Genotype status of the dopamine-related catechol-O-methyltransferase (COMT) gene corresponds with desirability of “unhealthy” foods. Appetite.

[53] Need AC, Ahmadi KR, Spector TD, Goldstein DB (2006). Obesity is associated with genetic variants that alter dopamine availability. Ann. Hum. Genet..

[54] Staley JR (2016). PhenoScanner: a database of human genotype-phenotype associations. Bioinforma..

[55] Stewart PM, Boulton A, Kumar S, Clark PM, Shackleton CH (1999). Cortisol metabolism in human obesity: impaired cortisone–>cortisol conversion in subjects with central adiposity. J. Clin. Endocrinol. Metab..

[56] Andrew R, Phillips DI, Walker BR (1998). Obesity and gender influence cortisol secretion and metabolism in man. J. Clin. Endocrinol. Metab..

[57] Pasquali R (1993). The hypothalamic-pituitary-adrenal axis in obese women with different patterns of body fat distribution. J. Clin. Endocrinol. Metab..

[58] Bujalska IJ, Kumar S, Stewart PM (1997). Does central obesity reflect “Cushing’s disease of the omentum”?. Lancet.

[59] Stimson RH (2009). Cortisol release from adipose tissue by 11beta-hydroxysteroid dehydrogenase type 1 in humans. Diabetes.

[60] Völzke H (2011). Cohort Profile: The Study of Health in Pomerania. Int. J. Epidemiol..

[61] National Cholesterol Education Program Expert Panel on Detection, E. & Treatment of High Blood Cholesterol in, A (2002). Third Report of the National Cholesterol Education Program (NCEP) Expert Panel on Detection, Evaluation, and Treatment of High Blood Cholesterol in Adults (Adult Treatment Panel III) final report. Circulation.

[62] IDF. *International Diabetes Federation: The IDF consensus worldwide definition of the metabolic syndrome*, http://www.idf.org/home/index.cfm?node=1429 (2005).

[63] Muller HP (2011). Quantification of human body fat tissue percentage by MRI. NMR Biomed..

[64] Knacke H (2016). Metabolic Fingerprints of Circulating IGF-1 and the IGF-1/IGFBP-3 Ratio: A Multifluid Metabolomics Study. J. Clin. Endocrinol. Metab..

[65] Piontek U (2017). Sex-specific metabolic profiles of androgens and its main binding protein SHBG in a middle aged population without diabetes. Sci. Rep..

[66] Benjamini Y, Hochberg Y (1995). Controlling the False Discovery Rate: A Practical and Powerful Approach to Multiple Testing. *Journal of the Royal Statistical Society*. Ser. B.

[67] Do KT (2015). Network-based approach for analyzing intra- and interfluid metabolite associations in human blood, urine, and saliva. J. Proteome Res..

